# Complete Genome Sequences of Five Phietaviruses Infecting Staphylococcus aureus

**DOI:** 10.1128/mra.00855-22

**Published:** 2022-09-29

**Authors:** Taylor P. Andrews, J. Steen Hoyer, Nicole L. Fahrenfeld, Jeffrey M. Boyd, Siobain Duffy

**Affiliations:** a Microbial Biology Graduate Program, Rutgers University, New Brunswick, New Jersey, USA; b Department of Ecology, Evolution, and Natural Resources, School of Environmental and Biological Sciences, Rutgers University, New Brunswick, New Jersey, USA; c Department of Civil and Environmental Engineering, School of Engineering, Rutgers University, Piscataway, New Jersey, USA; d Department of Biochemistry and Microbiology, School of Environmental and Biological Sciences, Rutgers University, New Brunswick, New Jersey, USA; Queens College CUNY

## Abstract

The annotated whole-genome sequences of five cultured phietaviruses infecting Staphylococcus aureus are presented. They are closely related to prophages that were previously sequenced as part of S. aureus genomes.

## ANNOUNCEMENT

Staphylococcus aureus is a human commensal bacterium that has the potential to cause life-threatening infection (1). Its interactions with bacteriophages are an increasingly studied part of microbiome studies (2). We present the annotated genomes of five plaque-purified S. aureus temperate phages in the genus *Phietavirus* (3). Four aliquots of municipal wastewater influent from a mid-Atlantic, U.S. treatment plant were collected in March 2021. To enrich for S. aureus phages, 5 mL of each sample was cocultured with S. aureus RN4220 (4) in tryptic soy broth (TSB) containing 10 mM CaCl_2_ (5). Phages were isolated using centrifugation and 0.22-μm filtration before being plated with S. aureus RN4220 using the pour-plate technique. Plaques underwent three rounds of subculturing through single plaques to yield purified phage stocks (6). The DNA genomes of five selected phages were extracted using QIAamp MinElute virus spin kits.

Paired-end (2 × 150-bp) sequencing using the Illumina DNA library preparation kit was performed on the NextSeq 2000 system at the Microbial Genome Sequencing Center (MiGS), which provided quality-controlled and trimmed reads. These reads were analyzed using the CPT Galaxy Phage Genome Assembler v2021.01 Workflow (https://cpt.tamu.edu/galaxy-pub) (7) with SPAdes v3.12.0 (http://cab.spbu.ru/software/spades) (8), which produced linear contigs with small overlaps at the end, suggesting that the genomes were circular. The overlaps were manually cut. Taxonomic assignment of the five double-stranded DNA (dsDNA) phage genomes was performed with GRAViTy v1.1.0 (http://gravity.cvr.gla.ac.uk) (9), which showed that they were phietaviruses (symmetrical Theil’s *U* value [reference prediction] of 0.863) related to SAP26 (GenBank accession number GU477322 [arbitrarily linearized]). The genomes were reoriented to reflect the termini of Staphylococcus prophages from a closely related genus (e.g., GenBank accession number DQ530359). Genome annotation was performed as described previously (10, 11); open reading frames (ORFs) were annotated using Prokka v1.14.6 (parameters Genus: *Phietavirus*, Kingdom: Viruses) in Galaxy (12) and further annotated for functionality with the PHROGs v4 database (https://phrogs.lmge.uca.fr) (13) and Phyre2 v2.0 (http://www.sbg.bio.ic.ac.uk/~phyre2/html/page.cgi?id=index) (14), and non-protein-coding features, including tRNAs (tRNAscan-SE v2.0) (http://trna.ucsc.edu/tRNAscan-SE) (15), terminators (ARNold v1.0) (http://rssf.i2bc.paris-saclay.fr/toolbox/arnold) (16), noncoding RNAs (Rfam v14.8) (https://rfam.xfam.org/search#tabview=tab1) (17), and promoters (Genome2D Prokaryote Promoter Prediction) (http://genome2d.molgenrug.nl/g2d_pepper_promoters.php) (18), were identified. Sequence coverage was calculated using Map with BWA-MEM v0.7.17.2 (19) and SAMtools Depth v1.13 in Galaxy (20). Default parameters were used except where otherwise noted.

**FIG 1:**
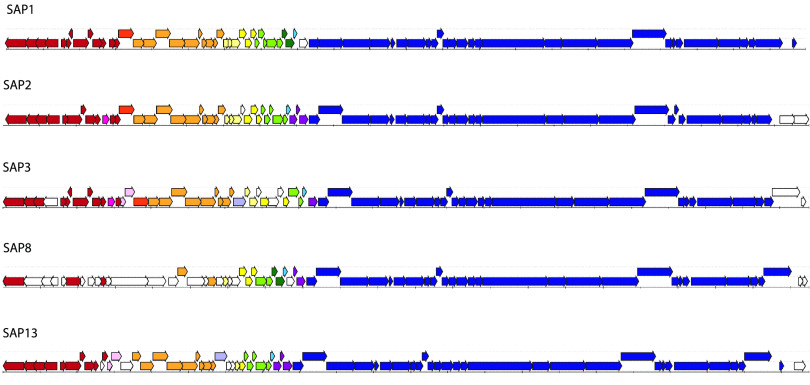
Genomic maps of the five phage genomes. Colors indicate blocks of homology, and ORFs without homology with other SAP genomes are depicted in white. All genomes have integrase genes at the 5′ end, indicating that they are likely capable of lysogeny. They share a large, syntenous block of genes toward the 3′ end, containing structural and hypothetical proteins.

The five SAP genomes are ~43 kb (Table 1), and portions of the genomes are very similar to one another (the most divergent pair, SAP1 and SAP8, are ≥94% identical by BLAST over 60% of the genome). There was significant synteny between the 63 to 69 ORFS of the genomes (Fig. 1). The closest BLAST hits to these phage genomes in the NCBI nonredundant database are all prophages within S. aureus genomes (e.g., SAP3 is 100% identical, with 100% query coverage, by BLAST to GenBank accession number CP051919).

**TABLE 1:** Summary of SAP phage genomic characteristics

Phage	Genome length (bp)	No. of predicted ORFs	No. of putative promoters	No. of putative rho-independent terminators	Avg sequencing coverage (×)	GC content (%)	No. of reads	GenBank accession no.	SRA accession no.
SAP1	43,962	68	10	22	9,518	34.3	2,896,630	ON911714	SRX16769400
SAP2	43,863	69	6	23	9,069	34.0	2,736,310	ON911715	SRX16769401
SAP3	43,586	66	11	18	11,412	34.6	3,405,310	ON911716	SRX16769402
SAP8	42,981	63	8	20	11,997	34.1	3,539,164	ON911717	SRX16769403
SAP13	43,478	67	10	25	11,145	34.6	3,316,128	ON911718	SRX16769404

### Data availability.

Genomes are available in GenBank (see Table 1 for the accession numbers). Illumina data are available in the NCBI SRA (BioProject accession number PRJNA857681) (Table 1). The phages are available by request from the corresponding authors.
